# Respiratory Muscle Strength in Healthy Children Aged 6 Years and Under: An Observational Study

**DOI:** 10.1002/ppul.71270

**Published:** 2025-09-16

**Authors:** Kayley Noxell, Emily Acquaye, Vicky MacBean

**Affiliations:** ^1^ Therapeutic Services Ltd Physiotherapy, St John's St. John's Newfoundland Canada; ^2^ Department of Health Sciences Brunel University of London Uxbridge UK

**Keywords:** child, infant, reference data, respiratory muscles

## Abstract

**Study Question:**

Measurement of respiratory muscle strength is important in the assessment and management of neuromuscular diseases. Reference data are essential for interpretation of clinical findings, but are lacking in infants and young children. This study aimed to provide reference data for maximum inspiratory (PImax) and maximum expiratory (PEmax) pressures in children aged 6 years and under.

**Materials and Methods:**

Healthy, term‐born children were eligible for inclusion. Age, height, weight and BMI were recorded, and height/weight/BMI‐for‐age percentiles calculated. PImax and PEmax were measured using a tight‐fitting face mask attached to a pressure transducer during maximal inspiratory and expiratory efforts respectively, induced via crying in younger participants and volitionally in older children. The greatest pressure was reported from three values within 20% of one another. Repeat measurements were obtained within a week where possible.

**Results:**

Sixty‐nine children aged 0.08–6.85 years were recruited, from whom technically‐acceptable PImax and PEmax data were obtained in 45 and 38 cases respectively. PImax was significantly and inversely related to age (Spearman's rho −0.339, *p* = 0.046); PEmax was not related to any anthropometric characteristics. Neither PImax or PEmax differed between male and female participants. Predicted PImax was 120 + (−3.89xage); mean (SD) PEmax was 80.3 (21.7) cmH_2_O. Repeatability coefficient was 17.2 cmH_2_O for PImax and 26.3 cmH_2_O for PEmax (based on eleven and nine children respectively).

**Answer to the Study Question:**

This study provides the first contiguous reference range from infancy through to school age. Reference data are provided for PImax and PEmax along with information on repeatability.

## 1 Introduction

Measurement of respiratory muscle strength is relevant to a number of clinical conditions seen in the pediatric population, most commonly neuromuscular conditions. Quantifying respiratory muscle strength in an individual patient and mapping trajectory over time will aid in monitoring disease progression and response to interventions; being able to compare measured to predicted values is essential if the magnitude of any impairment is to be assessed. With the advent of new treatments for neuromuscular conditions such as X‐linked myotubular myopathy [1], Duchenne muscular dystrophy [2] and spinal muscular atrophy [3], there is now a need to be able to quantify improvement as well as deterioration, and to determine when a child may have reached “normal” respiratory muscle strength.

Measurement of respiratory muscle strength involves inspiratory and/or expiratory efforts against an occluded airway, and hence can be uncomfortable even for cooperative adults who are able to understand the purpose of the test. In infants and very young children, crying is used to elicit a maximal inspiratory or expiratory effort, which is naturally distressing for the participant and potentially for parents/caregivers. In children beyond infancy but younger than school age, there is a transitional period in terms of development which may make testing more challenging. Children are unable to fully understand instructions and therefore cannot make reliable volitional efforts, but may not cry as infants do, hence maximal effort may be difficult to elicit.

Reference values are available for inspiratory and expiratory muscle strength in various pediatric populations, but there are some gaps. Most studies have either measured infants and very young children, or older children. Two recent systematic reviews and meta‐analyses [4, 5] have summarized the existing reference data available for respiratory strength testing in children, showing that most previous studies have been conducted in ages 7 and over, with a small number of studies including children as young as 4. Other studies have examined respiratory muscle strength in infants [6, 7], and one study assessed inspiratory and expiratory muscle strength from ages 0 to 3.76 years [8]. There is therefore a need for contiguous data across the age from infancy to early school‐age.

The aim of this study was to assess respiratory muscle strength through measurement of maximal inspiratory and expiratory pressures (PImax and PEmax) in a cohort of healthy infants and children under the age of 7 years. Doing so would produce reference values for the 0–6 age range. Secondary aims were to determine inter‐occasion repeatability of the tests and to explore success rates of testing across this age range.

## 2 Methods

This study was a cross‐sectional, observational study. The study received ethical approval from the College of Health, Medicine and Life Sciences Research Ethics Committee at Brunel University of London (reference 14043‐MHR‐Mar/2019‐18226). All procedures complied with the principles of the Declaration of Helsinki. Informed written consent was obtained from a parent or legal guardian of each participant. A shopping voucher (value 40 GBP) was issued to all participants in recognition of the time and inconvenience associated with participation. Testing was undertaken at Brunel University of London between October 2019 and September 2022, with a prolonged study hiatus between February 2020 and July 2022 due to restrictions imposed by the COVID‐19 pandemic.

Participants were recruited via social media advertising on local sites, posters around the local area, and word of mouth. Parents/guardians were able to read the participant information sheet and access a video showing the testing procedures before arranging an appointment.

### 2.1 Eligibility Criteria

Children were eligible for inclusion if they had been born at term (at least 37 weeks completed gestational age) and were aged 6 years of age or under at the time of testing. Children were excluded if they had a history of clinically significant gastrointestinal, renal, cardiovascular, hepatic, metabolic, allergic, dermatologic, hematologic, pulmonary, neurological, or psychiatric illness or disorder, or any clinically significant abnormalities of vital signs or clinical laboratory results (as assessed by the investigator during screening or on the day of testing, or reported by the parent/caregiver).

### 2.2 Anthropometrics

Standing height was measured using a calibrated stadiometer for children over the age of 2 years, or in supine with a measuring tape for children aged under 2, both with a resolution of 1 mm [9]. Weight was measured using a weighing scale with a resolution of 50 g, or in infants a weight from within the last week made by a healthcare professional. Weight/length‐, height‐ and BMI‐for‐age z‐scores were calculated using the WHO Anthro for personal computers (version 3.2.2) software package [10].

### 2.3 Respiratory Muscle Strength

Respiratory muscle pressure generation was measured using a face mask (8900 series, Hans Rudolph Inc., Shawnee, KS) attached to a one‐way valve (1240 series, Hans Rudolph Inc.) and differential pressure transducer (RMK1, GM Instruments, Irvine, Scotland, range +/−250 cmH_2_O). A pneumotachograph (4700 series, Hans Rudolph Inc.) attached to a differential pressure transducer (Spirometer, ADInstruments, Sydney, Australia) was also incorporated into the system to confirm unidirectional flow during the occlusion maneuvers. Pressure and flow signals were digitized (PowerLab 4/35, ADInstruments) and displayed on LabChart Pro software (version 8.2, ADInstruments). Both flow and pressure were calibrated using known reference signals before each testing occasion.

The face mask apparatus was placed over the child's nose and mouth for a minimum of eight respiratory effort cycles, until a plateau in pressure generation was observed (or until the child became too distressed to continue). One cycle was defined as a clear positive or negative pressure deflection (for expiratory and inspiratory testing respectively) followed by a return to atmospheric pressure. Older children received strong verbal encouragement to make maximal breathing efforts and were able to observe the pressure trace on the LabChart software to provide further motivation.

At least five of both inspiratory and expiratory maneuvers were performed, unless the child or parent/guardian indicated a wish to cease testing. Inspiratory maneuvers were generally performed first but, in some cases, PEmax was performed first as this test is often found to be less uncomfortable. Lack of success in inspiratory testing did not preclude attempts at expiratory testing and vice versa.

PImax and PEmax were determined from the maximal negative or positive pressure deflection respectively from the highest of three maneuvers within 20% of one another [11].

All children were invited to return for a repeat testing session within one week. Follow‐up sessions involved confirmation that no new illnesses had developed since the initial appointment, then repetition of respiratory strength testing (anthropometric measurements were not repeated). Both inspiratory and expiratory testing were attempted during follow‐up appointments even if unsuccessful during the initial session.

### 2.4 Sample Size and Statistical Analysis

No formal sample size calculation was undertaken; a convenience sampling approach was used with the aim to obtain as large a sample as possible within the timeframe of the study.

Distribution of data was tested for normality using the Shapiro Wilk test. Between‐group comparisons were assessed using unpaired *t* tests or Mann Whitney U tests for normally‐ and non‐normally distributed data respectively. Wilcoxon's matched pairs test was used to assess for any difference between strength measurements made on the two testing sessions. Relationships between variables were evaluated using a Pearson's or Spearman's correlation coefficient for normally‐ and non‐normally distributed data respectively. Chi‐square analysis was used to compare distribution of testing success rates across age groupings. Repeatability coefficient was calculated as described by [12]. A *p* value of < 0.05 was considered significant. All analyses were undertaken with SPSS version 29 for Windows (IBM, Chicago, IL).

## 3 Results

A total of 69 children were enrolled into the study. PImax and PEmax maneuvers were attempted in all participants. Four children were unable to perform three or more PImax maneuvers (of any quality), and 12 were unable to perform at least three PEmax maneuvers. Reasons for lack of success were refusal from the child (in older age groups) or parent/caregiver unwillingness to continue with testing due to visible distress in the child.

Technically acceptable values (three values within 20%) for PImax and PEmax were obtained in 45 and 38 children respectively.

Repeat visits were made by 23 children, with 11 and nine providing technically‐acceptable PImax and PEmax data respectively on both occasions. Some children did not provide acceptable data on the initial visit but were successful on the second occasion. Such data have been treated as successful data for visit one in data analysis.

Participant characteristics for the whole cohort and for those providing technically‐acceptable PImax and PEmax data on one and both visits are shown in Table 1. Youngest age was 0.08 years and oldest 6.85 years. Normality testing showed a significant deviation from a normal distribution for age, height, and BMI in the entire cohort.

**TABLE 1:** Participant characteristics.

	All participants (*n* = 69)	PImax successful on visit one (*n* = 45)	PEmax successful on visit one (*n* = 38)	Children providing repeat visit PImax (*n* = 11)	Children providing repeat visit PEmax (*n* = 9)
Age (years)	3.54 (1.82–5.07)	3.32 (1.43–5.45)	4.39 (1.74–5.54)	3.92 (0.97–5.82)	5.15 (4.32–5.70)
Sex (M:F)	43:26	31:14	29:9	7:4	8:1
Height (cm)	95.5 (81.5–113.5)	95.0 (78.8–115.0)	106.2 (83.0–115.0)	101.5 (69.0–121.5)	116.0 (107.5–120.8)
Weight (kg)	15.0 (4.87)	14.0 (10.0–20.0)	17.3 (11.0–20.0)	20.0 (7.5–20.0)	20.0 (17.0–20.0)
BMI (kg/m^2^)	15.2 (14.2–16.1)	15.3 (14.6–16.4)	15.0 (14.1–16.1)	15.1 (14.7–18.1)	14.9 (13.7–15.7)
Height‐for‐age z‐score	0.01 (0.92)	0.90 (−0.92 to 1.09)	0.19 (−0.92 to 1.12)	0.29 (−0.92 to 0.88)	1.12 (−0.01 to 1.94)
Weight‐for‐age z‐score	−0.07 (0.92)	−0.04 (−0.73 to 0.68)	−0.02 (−0.54 to 0.71)	0.00 (−0.73 to 0.91)	0.28 (0.05–0.90)
BMI‐for‐age z‐score	−0.14 (1.30)	−0.14 (−0.98 to 0.59)	−0.26 (−0.97 to 0.40)	−0.21 (−1.47 to 1.35)	−0.33 (−1.27 to 0.35)

*Note:* Data are presented as mean (SD), median (IQR) or counts.

Abbreviations: BMI, body mass index; cm, centimetres; F, female; kg, kilograms; M, male; PEmax, maximum expiratory pressure; PImax, maximum inspiratory pressure.

PImax showed a significant negative correlation with age (Spearman's rho −0.339, *p* = 0.046) but not with height, weight, BMI, or height‐, weight‐ or BMI‐for‐age z score. PEmax was not significantly related to age or to any anthropometric variables.

Median (IQR) PImax was not significantly different between male (115.9 (88.9–130.8) cmH_2_O) and female (122.5 (113.3–136.1) cmH_2_O) participants (*p* = 0.17). PEmax was also not significantly different between male (87.1 (55.6–112.3) cmH_2_O) and female (87.9 (68.3–120.9) cmH_2_O) participants (*p* = 0.827).

**FIGURE 1:**
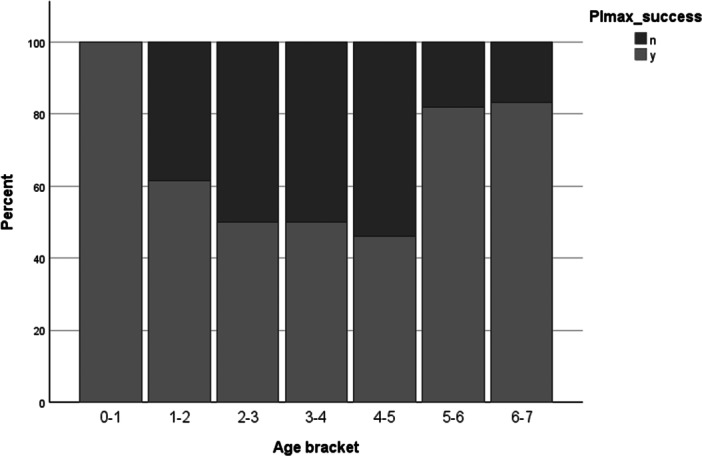
Percentage success rates of PImax across age groupings.

**FIGURE 2:**
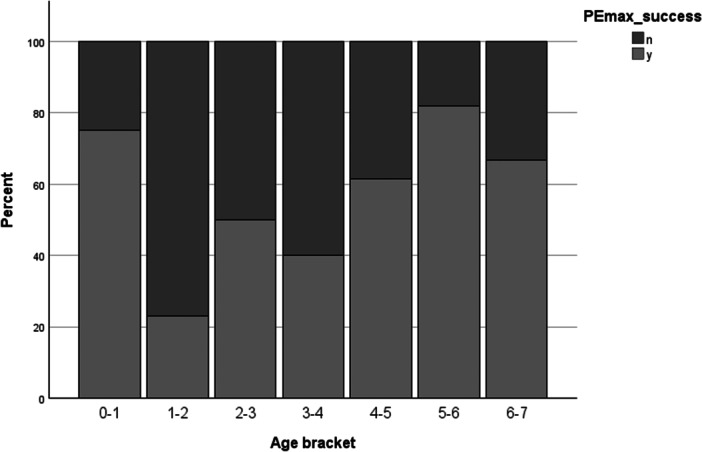
Percentage success rates of PEmax across age groupings.

The mean (SD) age of those who did (3.45 (2.08) years) and did not (3.55 (1.50) years) successfully produce technically‐acceptable (three values within 20%) PImax values was not significantly different (*p* = 0.835). The same was true for PEmax: mean (SD) age in the successful group was 3.81 (2.02) years and in the unsuccessful group 3.07 (1.66) years (*p* = 0.108). Distribution of testing success versus failure rates by age were not statistically significantly different to expected on Chi‐square testing (PImax *p* = 0.106; PEmax *p* = 0.77). When participants were divided into one year age bins and success/failure rates across age groupings plotted on a histogram, it can be seen however that there is an alinear relationship between age and likelihood of testing success. Higher success rates were seen in infants for both PImax and PEmax, lower success rates in the 1–4 age range, and increasing again once children reach school age (Figures 1 and 2).

There was no significant difference between PImax or PEmax measurements made on two occasions (*p* = 0.594 and *p* = 0.26 respectively). The repeatability coefficient was calculated as 17.2 cmH_2_O for PImax and 26.3 cmH_2_O for PEmax.

### 3.1 Predicted Values for Respiratory Muscle Strength

Based on the significant relationship observed with age, the equation to predict PImax (in cmH_2_O) in children aged six and under is:

$$\mathrm{PI}\max\;(\mathrm{cm}H_{2}O)=120.8+(-3.89\;*\;\mathrm{age in years})$$

As PEmax was not related to age or anthropometric characteristics, predicted PEmax is based on mean +/− 1.65 *SD. This gives a predicted PEmax of 80.3 cmH_2_O, with an SD of 21.7 cmH_2_O and a lower limit of normal (LLN) of 35.8 cmH_2_O.

## 4 Discussion

The current study provides reference data for measures of inspiratory and expiratory muscle strength in infants and children under the age of seven years. To date, no other authors have presented contiguous data across this age range. These data also suggest an alinear relationship between age and likelihood of testing success, with rates lower in those aged 1–4 years of age than in infants and early school‐aged children.

### 4.1 Comparison to Previous Data

As stated previously, there are limited data against which to compare the findings from the current study. Most applicable are the data from Shardonovsky et al. [8], who reported a mean (SD) for PImax of 118 (21) cmH_2_O and for PEmax 125 (35) cmH_2_O. Using the equation from the current study for PImax, the predicted inspiratory muscle strength for a 2‐year‐old (the midpoint of Shardonovsky's age range) would be 113.02 cmH_2_O, showing very close agreement. The PEmax data are slightly less well aligned, with the predicted values from the current study considerably lower (mean (SD) 80.3 (21.7) cmH_2_O).

Previous data obtained in cohorts of infants only show some variation between studies. Dimitrou et al. [6] reported a mean PImax of 70 cmH_2_O in healthy newborns, and Kassim et al [7] a slightly higher value of 88.8 cmH_2_O. Both are lower than the predicted value in the current study, though Kassim et al. also reported follow‐up values, with a mean PImax at six weeks postnatal age of 100.9 cmH_2_O. PEmax from these two previous studies are also lower than those found in the current work, with mean PEmax values of 53 cmH_2_O and 61.8 cmH_2_O [6, 7]. Once again however, six week follow‐up data from the Kassim et al. study showed higher values (mean PEmax 82.6 cmH_2_O), which align well with the findings of the current work. The substantial and rapid maturational changes occurring immediately after birth are likely to underlie the differences between the predicted values from the current study and those previously devised from newborn infants.

The systematic review and meta‐analysis conducted by Verma et al. [4] devised sex‐specific prediction equations for PImax and PEmax based on data from 3509 children aged 4–18. Their equations incorporated age and weight and/or height, though these parameters still only explained less than 25% of the variance in strength. This suggests that there are other intrinsic factors than influence respiratory muscle strength in children. A more recent and larger systematic review and meta‐analysis [5], incorporating data from almost 6000 children aged 4–19 did not produce composite reference equations, but did provide sex‐specific mean values across two age groups (4–11 and 12–19). All predicted values were lower than those devised from the current study, with the exception of predicted PEmax in boys (mean [95% confidence interval] 84.0 (73.6–94.3) cmH_2_O).

We did not observe sex differences in respiratory muscle strength values in the current study. The fact that other studies in older children have seen such differences is perhaps unsurprising, as many of these studies included peri‐ and postpubertal participants where sex hormones are expected to exert a greater influence over muscle strength and body composition.

The only finding from the current study that has not been demonstrated in previous work is the negative relationship between PImax and age. This was a weak correlation (rho = −0.339) and can perhaps be explained by the fact that testing quality appeared higher in the youngest ages (100% success rate in 0–1 year age group). When harnessing purely autonomic respiratory drive, “true” maxima are most likely to be obtained, so this relationship may be more related to the testing approach than to physiological changes. Nevertheless, the same would be observed in clinical testing, so the reference equation remains valid. Replication of this finding in future studies would be of value.

### 4.2 Practical Considerations for Testing

Inspiratory muscle testing is anecdotally perceived to be more unpleasant than expiratory muscle testing, perhaps because effortful, resisted expiration is a more familiar maneuver encountered in everyday life (e.g., Valsalva maneuver when straining at stool or lifting heavy objects). Despite this, we showed a slightly higher success rate in PImax than PEmax testing. This may in part reflect the physics of each testing procedure: in PImax testing the negative pressure generated “sucks” the mask onto the participant's face, minimizing loss of pressure. When performing PEmax, the positive pressure increases the chance of leak and therefore variability between maneuvers. During testing, we also prioritized PImax testing as this is more commonly used clinically, so some children (and/or parents) may have tired of the testing once reaching the PEmax maneuvers.

When testing volunteer, healthy children in a research context it is essential to observe robust ethical principles. The participants were not gaining any direct personal benefit from the testing (their efforts were recognized with a monetary voucher, but this was not contingent on their producing technically‐acceptable data). We therefore adopted a lower threshold for refusal or discontinuation of testing than may be used in a clinical setting, where test results are essential to guide assessment and/or management of serious medical conditions. Testing success rates greater than the 68% and 55% observed in this study for PImax and PEmax respectively may be seen in clinical practice. Indeed, in a longitudinal observational study of children aged under four living with neuromuscular disease [13], PImax and PEmax were successfully measured in all 34 children, and repeated measures obtained over a period of up to 30 months (see Figure 2B in Dowling et al. [12]). Similar experiences were obtained from an interventional study in a comparable population [1]. Anecdotally, the unpleasantness of the testing appears greater in stronger participants (perhaps due to sensory stimulation being proportionate to intrathoracic pressure generation). No studies to date have explored child and/or parental experience of such testing, and qualitative data would be of interest in this field. Quantitative data on attendance/testing success rates in clinical populations would also be informative.

Of note, the relatively low testing success rates also reflect close attention to data quality. International guidelines require three maneuvers within 20% of one another to maximize the likelihood that results reflect “true” strength (similar to other maximal tests of respiratory function such as spirometry). Adherence to these standards in clinical practice is essential to ensure reliable data. Using data that do not meet reproducibility criteria may under‐estimate a child's strength (they may be able to perform better than that which could be measured), but will never over‐estimate, meaning that clinical deterioration cannot be missed.

### 4.3 Strengths and Limitations

Our study is the first to assess respiratory muscle strength in a contiguous sample of healthy children across the age range from 0 to 6.99 years, and therefore has value to clinicians and researchers working with children in this age range affected by neuromuscular disorders. Comparison to reference data is essential for quantifying disease severity and response to treatment. While our sample size is only moderate in size, these data still add substantially to the field.

A key consideration in our study was the choice of testing methodology for use across the cohort. Children aged four years of age and above would potentially be capable of performing the tests using the same methods adopted in older children and adults, namely volitional and sustained efforts via a mouthpiece [11]. However, this results in a discontinuous data set, making results around the transition from one testing approach to the next difficult to interpret. Children will also develop their ability to transition from one testing method to the other at different ages. By developing reference data that overlap with those derived from “adult” testing methods, is it possible to ensure that children being monitored longitudinally do not have breakpoints in the reference data used to support their care.

A clear limitation of the current study is the low number of children who returned for repeat testing, and the low proportion of those children who provided technically‐acceptable repeat data. This particular form of testing is arduous and quite unpleasant for many children, and for their parents/caregivers who observe the testing. As stated above, testing success rates are influenced in this study by ethical considerations. With no direct benefit of the testing, we were not expecting a high return rate, but the low numbers of successful repeat visits observed are nonetheless disappointing. Willingness to return is likely to have been influenced by both the testing procedure itself as well as more pragmatic considerations such as parental work schedules and convenience of attending, though we did not collect data on reasons for declining to return. We have been able to generate data on the repeatability coefficient of PImax and PEmax, but recognize that these values are not robust. With a larger data set from future studies, narrower limits for inter‐occasion repeatability of the tests would be likely.

## 5 Conclusion

This study has expanded the available reference data for inspiratory and expiratory muscle strength in infants and children under the age of seven. The availability of a contiguous data set throughout this age range, overlapping with those developed in children of school age using different methodology, facilitates longitudinal interpretation of respiratory strength data in research and clinical practice. Limited data are presented for repeatability coefficients; further studies are required to expand on these latter findings.

## Author Contributions

**Kayley Noxell:** writing – review and editing, formal analysis, project administration, data curation, investigation. **Emily Acquaye:** investigation, writing – review and editing, project administration, data curation. **Vicky MacBean:** conceptualization, investigation, funding acquisition, writing – original draft, methodology, writing – review and editing, formal analysis, project administration, data curation, supervision.

## Conflicts of Interest

This study was funded by Astellas Gene Therapies (previously Audentes Therapeutics Inc), including provision of study equipment. The company had no influence on the study design or interpretation of results. Vicky MacBean has previously received institutional and personal fees from Astellas Gene Therapies and Audentes Therapeutics. Kayley Noxell and Emily Acquaye have no conflicts of interest to declare.

## Data Availability

The data that support the findings of this study are openly available in FigShare at https://brunel.figshare.com/, reference number 10.17633/rd.brunel.29651501. Study data are available at https://doi.org/10.17633/rd.brunel.29651501.
